# Advances in the Discovery of Efflux Pump Inhibitors as Novel Potentiators to Control Antimicrobial-Resistant Pathogens

**DOI:** 10.3390/antibiotics12091417

**Published:** 2023-09-07

**Authors:** Song Zhang, Jun Wang, Juhee Ahn

**Affiliations:** 1 Department of Biomedical Science, Kangwon National University, Chuncheon 24341, Republic of Korea; ed5988449@kangwon.ac.kr; 2 College of Food Science and Engineering, Qingdao Agricultural University, Qingdao 266109, China; 3 Institute of Bioscience and Biotechnology, Kangwon National University, Chuncheon 24341, Republic of Korea

**Keywords:** multidrug efflux pump, antibiotic resistance, efflux pump inhibitor, biofilm formation, combination therapy

## Abstract

The excessive use of antibiotics has led to the emergence of multidrug-resistant (MDR) pathogens in clinical settings and food-producing animals, posing significant challenges to clinical management and food control. Over the past few decades, the discovery of antimicrobials has slowed down, leading to a lack of treatment options for clinical infectious diseases and foodborne illnesses. Given the increasing prevalence of antibiotic resistance and the limited availability of effective antibiotics, the discovery of novel antibiotic potentiators may prove useful for the treatment of bacterial infections. The application of antibiotics combined with antibiotic potentiators has demonstrated successful outcomes in bench-scale experiments and clinical settings. For instance, the use of efflux pump inhibitors (EPIs) in combination with antibiotics showed effective inhibition of MDR pathogens. Thus, this review aims to enable the possibility of using novel EPIs as potential adjuvants to effectively control MDR pathogens. Specifically, it provides a comprehensive summary of the advances in novel EPI discovery and the underlying mechanisms that restore antimicrobial activity. In addition, we also characterize plant-derived EPIs as novel potentiators. This review provides insights into current challenges and potential strategies for future advancements in fighting antibiotic resistance.

## 1. Introduction

Since the first discovery of penicillin in 1928, antibiotics have revolutionized modern medicine for treating bacterial infections in food-producing animals and humans. However, the overuse and misuse of antibiotics have led to the development of antibiotic resistance in bacteria, resulting in serious public health problems [1]. The rapid emergence and dissemination of antibiotic resistance have limited chemotherapeutic options. Furthermore, the infections caused by multidrug-resistant (MDR) bacteria have increased the risk of treatment failure due to the lack of effective antibiotics. The antibiotic resistance mechanisms in bacteria include the production of antibiotic-hydrolyzing enzymes, activation of efflux pumps, modification in targeting sites, reduction in membrane permeability, and development of alternative metabolic bypass [2]. Among these, the activation of efflux pumps is one of the major acquired antibiotic resistance mechanisms that can lead to the development of MDR pathogens [3]. Efflux pumps are bacterial membrane transporters that facilitate the active translocation of substrates such as antibiotics, dyes, metabolites, quorum-sensing signals, and virulence factors [4]. Commonly, MDR bacteria possess multiple efflux pumps to expel antimicrobial agents.

Efflux pumps are capable of recognizing and delivering specific substrates with high affinity, which can be called substrate specificity [5]. This property enables bacteria to utilize their efflux pumps, which can recognize and expel antimicrobial agents, to reduce the intracellular concentration of drugs and develop antimicrobial resistance. For example, *Staphylococcus aureus* can utilize the MepA efflux pump to extrude chlorhexidine, cetrimide, and dequalinium [6]. *Salmonella enteritica* can expel norfloxacin, doxorubicin, and acriflavine by the use of the MdtK efflux pump [7]. Current studies show that many factors can activate the efflux pumps and facilitate the development of MDR pathogens, such as environmental signals, regulatory proteins, and multiple efflux-pump-associated gene mutations. In terms of environmental signals, lincomycin and boric acid have been found to induce the activation of efflux pumps and promote transiently reduced susceptibility to antibiotics in *Stenotrophomonas maltophilia* [8]. The involvement of regulatory proteins, including RamA, SoxS, and RobA, has also been proven to influence the activation of the efflux pump in *Enterobacter cloacae* [9]. Furthermore, the MDR phenotype observed in *Pseudomonas aeruginosa* was attributed to the simultaneous overexpression of the efflux-pump-associated genes, *mexA* and *mexXY* [10]. Overall, bacteria can employ multiple mechanisms to activate the efflux pumps, thereby augmenting their resistance to various antimicrobial agents. Therefore, there is a strong relation between the activation of efflux pumps and the formation of MDR pathogens. It may provide a useful therapeutic approach to overcome antimicrobial resistance by impeding or bypassing the efflux pumps in the course of their duty.

Alternative methods to bypass the efflux pumps have been used to control MDR bacterial infections, including antibiotic cycling and antibiotic combinations [11]. Antibiotic cycling is used to reduce antibiotic resistance and preserve antibiotic activity through sequential treatments [12]. However, antibiotic cycling cannot eradicate the MDR pathogens through the periodic replacement of antibiotics because of the repeated selection pressure on bacteria and the development of antibiotic resistance [13]. Antibiotic combinations have also been utilized to overcome antibiotic resistance by combining two or more different classes of antibiotics. Pathogens are required to acquire more than two subsequent mutations to develop resistance, which can lead to increased fitness costs and decreased survival rates of MDR bacteria. Nevertheless, mixed antibiotics may exhibit complicated interactions between antibiotics and unmatched pharmacokinetics, ultimately making it difficult to predict synergistic antimicrobial effects [14]. Efflux pump inhibitors (EPIs) can interact with antibiotics and inhibit efflux pumps that maintain a high concentration of antibiotics in bacteria. Specifically, EPIs can disrupt the function of efflux pumps, suppressing the extrusion of antibiotics and leading to enhanced susceptibility of bacteria to various antibiotics. Thus, the application of EPIs can be a promising approach to control MDR bacterial infections.

Currently, EPIs are increasingly used in laboratories to assess compatibility in clinical applications and understand their mechanism of action. EPIs disrupt the function of efflux pumps through one or multiple mechanisms. These mechanisms primarily involve obstructing the energy supply to efflux pump systems, preventing substrates from binding to active sites of efflux pumps, and downregulating the gene expression of efflux pumps [15]. For example, carbonyl cyanide-m-chlorophenylhydrazone (CCCP) has the ability to disrupt the proton motive force (PMF) and consequently inhibit the activity of efflux pumps [16]. Moreover, phenylalanyl arginyl β-naphthylamide (PAβN) can function as a competitive inhibitor of substrate binding, which can impede antibiotic efflux in MDR bacteria [17]. However, the nephrotoxicity of PAβN and the oxidative stress caused by CCCP seem to be excessively toxic in clinical practice [18,19]. In recent years, numerous natural compounds have been reported to possess efflux pump inhibitory with less toxicity. Therefore, it may be applicable to explore natural compounds for the discovery of potential EPIs. This review discusses the newly identified synthetic and natural EPIs and highlights the possibility of using EPIs to effectively control MDR pathogens.

## 2. Classification of Efflux Pumps and Their Roles in Antibiotic Resistance

**Figure 1:**
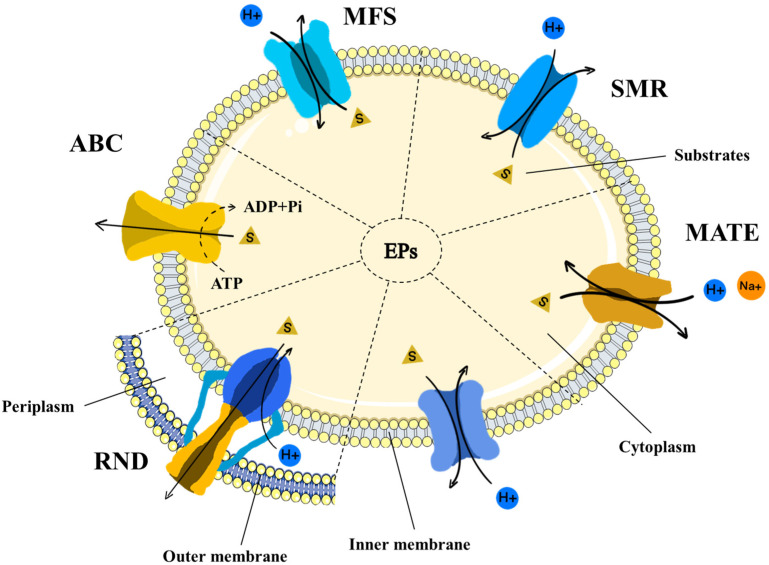
The main efflux pump systems in pathogens, including the adenosine–triphosphate (ATP)-binding cassette superfamily (ABC), the major facilitator superfamily (MFS), the multidrug and toxic compound extrusion family (MATE), the resistance–nodulation–cell division superfamily (RND), the small multidrug resistance family (SMR), and the proteobacterial antimicrobial compound efflux family (PACE). The efflux pumps in Gram-negative bacteria comprise the inner membrane transporters, the periplasmic adapter proteins, and the outer membrane channel proteins. ABC utilizes energy derived from ATP hydrolysis. The secondary active transporters acquire the energy stored in ion gradients (H^+^ or Na^+^). ADP: adenosine diphosphate; Pi: inorganic phosphate; EPs: efflux pumps, S: substrate.

Based on the substrate properties, coupling energy, and transporter structures, efflux pumps have been classified into six families, namely the adenosine–triphosphate (ATP)-binding cassette superfamily (ABC), the major facilitator superfamily (MFS), the multidrug and toxic compound extrusion family (MATE), the resistance–nodulation–cell division superfamily (RND), the small multidrug resistance family (SMR), and the proteobacterial antimicrobial compound efflux family (PACE) [20,21,22]. Among these efflux pumps, the ABC family, classified as primary active transporters, facilitates the movement of antibacterial agents across the membrane through the acquisition of energy via ATP hydrolysis [23]. In contrast, the other five families, categorized as secondary active transporters, utilize the energy stored in ion gradients to expel their substrates [24]. The efflux pumps have been well developed in various Gram-positive and Gram-negative bacteria [3]. In Gram-positive bacteria, efflux pumps exhibit as single-component transporters located at the cytoplasmic membrane. In Gram-negative bacteria, efflux pumps are multiple-component systems, also known as the trimer, synergistically responsible for the extrusion of antibiotics [25] (Figure 1 and Table 1).

The ABC superfamily is composed of three main categories: importers responsible for transporting amino acids, metals, ions, and other substances; exporters that facilitate the extrusion of toxins, antibiotic agents, and polysaccharides; and a final type that participates in DNA repair or translation [50]. Many ABC efflux pumps have been described as multidrug transporters such as MacAB-TolC in *Escherichia coli*, LmrA in *Lactococcus lactis*, EfrAB in *Enterococcus faecalis*, and PATA/B in *Streptococcus pneumoniae*. These efflux pumps can transport macrolides, lincosamides, hydrophilic fluoroquinolones, aminoglycosides, chloramphenicol, and disinfectants across the membrane [51].

The MFS efflux pumps constitute the most extensive secondary transporter family, encompassing over 10,000 sequenced members. These efflux pumps mainly transport sugars while some MFS transporters also participate in the efflux of drugs, thus potentiating antibiotic resistance [52]. These transporters are widely expressed in various MDR pathogens, specifically in Gram-positive bacteria. MFS efflux pumps such as NorA and Tet38 in *Staphylococcus aureus*, LmrP in *L. lactis*, and KpnGH in *Klebsiella pneumoniae* have been well characterized to mediate the resistance to fluoroquinolones, tetracyclines, streptogramins, macrolides, lincosamides, and detergents [53].

The MATE transporters are mainly classified into three distinct types, namely NorM, DNA damage-inducible protein F (DinF), and eukaryotic subfamilies [54]. NorM is capable of mitigating oxidative stress damage by exporting intracellular reactive oxygen [55], while DinF can effectively reverse susceptibility to moxifloxacin, ciprofloxacin, and levofloxacin [56]. The most common MATE transporters include PmpM in *P. aeruginosa*, and MepA and NorM in *S. aureus* [57]. Unlike other secondary active transporters, MATE efflux pumps can utilize Na^+^ and H^+^ as the driving force to confer resistance to various substrates such as tigecycline, hydrophilic fluoroquinolones, dyes, and fungicides [43].

The RND efflux pumps comprise inner membrane transporters, periplasmic adapter proteins, and outer membrane channel proteins [58]. Specifically, the substrates in the cytoplasm can be located and transported to the periplasmic space or the outer leaflet of the cell membrane, intercepted by periplasmic adapter proteins, and ultimately expelled to the exterior through the channel proteins [51]. RND superfamily members are the predominant transporters in Gram-negative bacteria and exhibit a broad substrate spectrum. RND transporters include AcrAB-TolC in *E. coli*, MexAB-oprM in *P. aeruginosa*, and AdeABC, AdeFGH, and AdeIJK in *A. baumannii* [49], which are extensively involved in the extrusion of various substances such as chloramphenicol, fluoroquinolones, novobiocin, tetracycline, organic solvents, and dyes [33,36]. Other RND efflux pumps have also been described in Gram-positive bacteria such as FarE, identified in *S. aureus*, which confers resistance to linoleic acid, fatty acid, and rhodomyrtone [37].

The SMR transporters are the smallest MDR efflux pumps and are mainly categorized into two physiological subtypes: (i) guanidinium exporter involved in the allocation of bacterial metabolites and (ii) quaternary ammonium compound representative subtype responsible for the extrusion of toxic compounds [51]. It has been elaborated that both subtypes work separately and do not interfere with each other [59]. Several SMR proteins have been identified in many pathogens and can endow with resistance to a broad range of antibiotics. For example, the most investigated EmrE pump in *E. coli* can confer resistance to various quaternary cation compounds and osmoprotectants [44]. Other transporters such as KpnEF in *K. pneumoniae*, QacC in *S. aureus*, and EbrAB in *Bacillus subtilis* have also been elaborated to alleviate susceptibility to various cationic lipophilic dyes and multiple antibiotics [45,48].

The PACE family is the recently discovered transport protein known as *Acinetobacter* chlorhexidine efflux protein I (AceI) from *A. baumannii* [60]. This efflux pump is primarily classified into two clades: the chlorhexidine-responsive clade and the chlorhexidine-unresponsive clade [22,61]. The chlorhexidine-responsive clade can utilize the electrochemical proton gradient as the primary energy source to expel substrates such as proflavine, chlorhexidine, acriflavine, dequalinium, and benzalkonium [62]. Many other homologous domain proteins of AceI have been unveiled in distinct pathogens including *K. pneumoniae*, *P. aeruginosa*, *Enterobacter*, and *Burkholderia* [63]. Recently, a new PACE transporter has also been identified as PA2889 in *P. aeruginosa*, which is capable of expelling chlorhexidine through the cell membrane [64].

## 3. Efflux-Pump-Mediated Biofilm Formation

Biofilms are known as the stable aggregation of bacteria encapsulated in the extracellular polymeric substances (EPSs), attached to abiotic and biotic surfaces [65]. In comparison with planktonic cells, biofilm cells can exhibit enhanced tolerance to antibiotic treatments, which may result from the diminished permeability of antibiotics to EPS matrix, reduced growth rate of biofilm bacteria, exchange of plasmids containing multidrug-resistant genes, and regulation of quorum signals [66,67]. Much evidence has been presented that efflux pumps are inextricably linked to dynamic biofilm formation. The suppression of efflux pumps such as *acrB*, *emrE*, and *mdtE* can inhibit biofilm formation in *E. coli* [68]. In *S. aureus*, the *mdeA*, *norB*, and *norC* genes were overexpressed in the process of biofilm formation [69]. Specifically, efflux pumps can mediate the mass transport of EPSs and the signaling molecules of the quorum sensing (QS) system to directly affect biofilm formation. In addition, efflux pumps can indirectly influence biofilm formation by regulating the expression of biofilm-associated genes [70] (Table 2).

EPSs mainly include polysaccharides, nucleic acids, lipids, and proteins, which contribute to the structural integrity of biofilms, effective adhesion to surfaces, and decreased diffusion of antimicrobials [65]. In addition, the EPS matrix plays an important role in storing metabolic substances and providing nutrients and energy for bacterial biofilms [79]. Efflux pumps are responsible for the transport of EPSs to facilitate biofilm formation. For instance, the upregulated MFS pump SetB in *E. coli* has been proven to extrude glucose to facilitate the synthesis of the EPS matrix [71]. The ABC pump YhdX can transport *L*-amino acids to the biofilm matrix, which will promote biofilm stability through electrostatic interactions with other molecules [72]. It has been reported that the MFS pump AraJ is in charge of the efflux of arabinose which can accelerate the aggregation of bacteria and the process of biofilm formation [65].

QS is an intercellular communication mechanism that allows bacteria to recognize the extracellular autoinducers (AIs) and regulate their gene expression in response to the changed environmental conditions [70]. At present, QS signals are divided into three types, namely autoinducing peptide (AIP) in Gram-positive bacteria, *N*-acyl homoserine lactones (AHLs) in Gram-negative bacteria, and autoinducer-2 (AI-2) in both Gram-positive and Gram-negative bacteria [66,80]. The participation of the QS system is crucial to the formation and maturation of biofilm formation in various pathogens. In *S. aureus*, AIP signals such as Agr are capable of regulating the dispersion of biofilm and the spread of biofilm-related infections [81]. The bacterial twitching motility and biofilm attachment have been reported to be influenced by AHL systems, which promote the integrity and stability of biofilm formation [82]. Furthermore, the AI-2 signals such as QseBC can upregulate the expression of biofilm-related genes such as *bcsA*, *fliC*, *fimA*, and *motA* to promote biofilm formation in *E. coli* [83]. Efflux pumps play a crucial role in the transport of QS signals to regulate biofilm formation. For example, MexAB-OprM, as the main efflux pump in *P. aeruginosa*, can transport *N*-3-oxododecanoyl-l-homoserine lactone out of the membrane and facilitate biofilm formation [73]. The downregulation of MsrA mediated by EPIs decreased the transcription levels of QS signals such as *agrA* and *sarA* and then inhibited the formation of biofilm [74]. Moreover, Lsr ABC transporters can deliver AI-2 signals and result in enhanced bacterial aggregation and adhesion [84]. Notably, previous studies reported that the overexpression of efflux pumps may inhibit the growth of biofilm in some cases. The concentration of QS signals in *P. aeruginosa* can be reduced by the overexpression of MexEF-OprN, resulting in diminished quorum response and impaired biofilm formation [75]. In *Acinetobacter baumannii*, the decreased formation of biofilm was observed due to the overexpression of the AdeABC, AdeFGH, and AdeIJK transporters [77]. Hence, it may be a promising strategy to investigate the regulation of QS systems mediated by efflux pumps and prevent the formation and diffusion of biofilm cells.

Many studies have identified the adherence-associated genes, which are closely relevant to biofilm formation in distinct aspects. In *S. aureus*, *icaABC* and *icaR* are mainly involved in the synthesis of capsular polysaccharide/adhesion (PS/A) and polysaccharide intercellular adhesin (PIA), which are essential to the production of biofilm [85]. In *K. pneumoniae*, the *mrkA*, *mrkD*, and *fimH* genes were observed to encode the multiple types of fimbrial adhesion involved in biofilm formation [86]. In recent years, efflux pumps have been demonstrated to be intimately linked to the expression of biofilm-associated genes. In *Listeria monocytogenes,* a new ABC transporter encoded by the *lm.G_1771* gene can negatively regulate the genes related to biofilm formation such as cell surface anchor proteins (SrtA), cell surface proteins (Dlt), and transcriptional regulators (GntR) [76]. The *csuA/B*, *csuC,* and *fimA* genes have been recognized as the biofilm-associated genes that are responsible for adhesion, colonization, and microcolony formation [77]. These genes were reported to be downregulated in the mutants overexpressing the efflux genes, including *adeABC* and *adeIJK*, resulting in diminished biofilm formation. Furthermore, it has been documented that the consistent upregulation of *adeG* and *abaI* encoding AHL synthases accelerated the synthesis and transport of AHLs, leading to the most extensive biofilm induction in *A. baumannii* [78].

**Figure 2:**
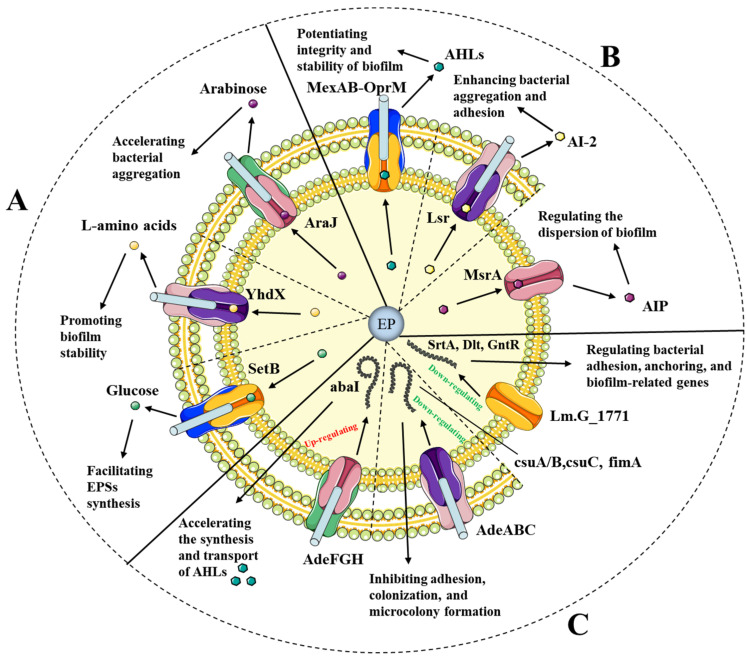
Diagram of biofilm formation mediated by representative efflux pumps and EPSs involved in biofilm formation (**A**), efflux pumps and QS signals involved in biofilm formation (**B**), and efflux pumps and biofilm-associated genes (**C**). AHLs, *N*-acyl homoserine lactones; AI-2, autoinducer-2; AIP, autoinducing peptide.

Overall, efflux pumps are closely involved in the process of biofilm formation, including the production and transport of EPSs, the regulation and transport of QS signals, and the regulation of biofilm-related genes. The EPSs and QS signals delivered by efflux pumps result in the accelerated aggregation of bacteria, augmented synthesis of EPSs, and enhanced integrity of biofilm. Moreover, the suppression of biofilm-related genes mediated by efflux pumps influences the attachment and formation of biofilm (Figure 2). Notably, the effects of efflux pumps on biofilm are variable depending on the different stages and parts [87]. As an illustration, the internal biofilm cells may be inclined to overexpress the efflux pumps associated with the extrusion of secondary metabolites, while the external bacteria may tend to activate the transporters involved in the resistance to antimicrobial agents. Therefore, it is crucial to thoroughly investigate the function and substrate spectrum of efflux pumps in biofilm formation and avoid the accidental induction of biofilm resulting from the misuse of EPIs and antimicrobial agents.

## 4. EPIs as a Promising Strategy to Combat Antimicrobial Resistance

EPIs can inhibit the function of efflux pumps through distinct mechanisms, reducing antibiotic resistance [5]. In light of the crucial role of efflux pumps in antibiotic resistance, the development of EPIs seems to be a promising and practical strategy for controlling MDR bacteria. Many studies have documented that the combination of EPIs and antibiotics effectively prevented the extrusion of antibiotics by efflux pumps and enhanced antimicrobial activity. For example, D13-9001 and MBX2319, as synthetic EPIs, can interact with MexAB-OprM and AcrAB-TolC, resulting in the increased accumulation of antibiotics in pathogens [88,89]. In addition, EPIs are able to eliminate the biofilm formation mediated by efflux pumps and decrease the antibiotic tolerance of biofilms. The addition of PAβN or thioridazine distinctly decreased biofilm formation by up to 80% [90]. However, the application of EPIs to prevent antibiotic resistance is still at an initial stage, which requires further study to identify successful EPIs for clinical utilization and animal-producing food products.

## 5. Sustainability Criteria for EPIs

**Figure 3:**
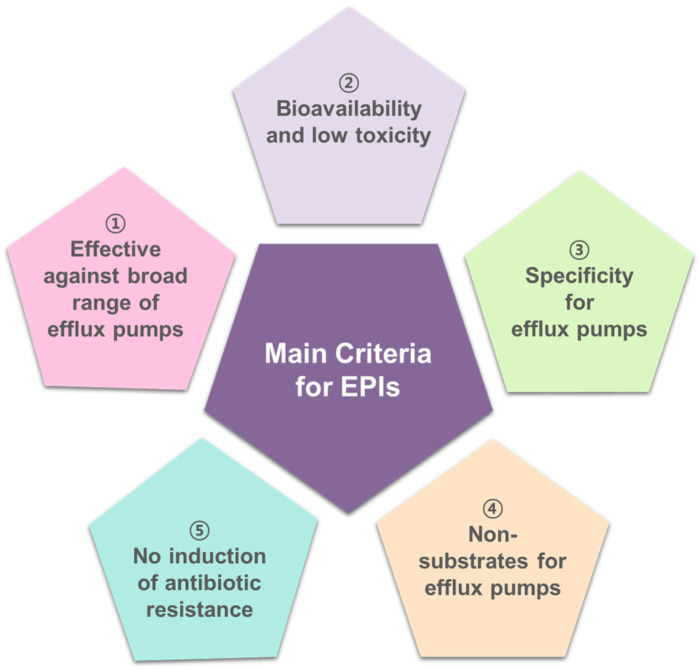
The main criteria for the discovery of efflux pump inhibitors (EPIs).

The EPI-associated compounds have been discovered to effectively inhibit efflux pumps in bacteria. Major criteria should be met for these compounds that can be considered excellent EPIs, including (1) broad-spectrum activity of EPIs against various efflux pumps, (2) no side effects and bioavailability for clinical use, (3) specific EPIs against efflux pump activity, (4) non-substrates to the binding sites of efflux pumps, and (5) prevention of antibiotic resistance development (Figure 3).

Many traditional EPIs have been extensively investigated to suppress antimicrobial resistance and restore antibiotic activity against pathogens. MBX2319 is a synthetic pyrazolopyridine capable of decreasing by 8-fold the MIC of ciprofloxacin, levofloxacin, and piperacillin against *E. coli* [89]. Similarly, it has been reported that piperine can restore the susceptibility of *S. aureus* to ciprofloxacin and lead to a 4-fold reduction in MIC [91]. Many compounds such as flavonoids and phenothiazines exhibited inhibitory effects on efflux pumps and antimicrobial activity [92,93,94]. However, the antimicrobial activity of EPIs is associated with the development of resistance. Therefore, EPIs with non-antimicrobial activity may show a long drug lifecycle in utilization. For instance, the 1,8-naphthyridines involved in the inhibition of efflux pumps have no antibacterial activity, resulting in a decrease in developing resistance to EPIs in bacteria [95]. Despite many compounds capable of enhancing antibiotic activity, it is still crucial to confirm the compounds mainly targeting the efflux pumps rather than other side mechanisms. PAβN is a broad-spectrum EPI that can induce an 8-fold decrease in the MIC of levofloxacin against *P. aeruginosa*, while a 64-fold reduction in MIC can be observed in *P. aeruginosa* overexpressing MexAB-OprM transporters [17]. It has been documented that NSC 60339 potentiated novobiocin and erythromycin in *E. coli*, but exhibited no effect on these antibiotics in *E. coli* lacking efflux pumps [96]. In contrast, three compounds, NSC56410, NSC 260594, and NSC 26980, can intensify antibiotics without the suppression of efflux transporters. The efflux-independent manners may imply the presence of other mechanisms that potentiate antibiotic activity against bacteria, while different mechanisms may exhibit latent off-target effects and induce cytotoxicity.

The current EPIs can be primarily classified into two types, including competitive substrate inhibitors and non-competitive substrate inhibitors. A study reported that geraniol can competitively bind to the AcrAB-TolC efflux pump and restore antibiotic susceptibility against *A. baumannii*, *E. coli*, *E. aerogenes*, and *P. aeruginosa* [97]. As a protoberberine alkaloid, columbamine has been demonstrated to interfere with ATP synthesis and impact the formation of proton electrochemical gradients, thereby impairing the transport of efflux pumps [98]. In general, competitive EPIs are substrates for the target transporters, which can induce the overexpression of efflux pumps and ultimately cause loss of inhibitory activity. Gram-negative bacteria are surrounded by the outer membrane which can function as a selective permeation barrier and protect bacteria from antimicrobial agents such as vancomycin, geranylamine, and MBX-4191 [30,99]. Antibiotic resistance in Gram-negative bacteria can be reversed by the EPIs which are capable of overcoming the outer membrane barrier. Previous studies have demonstrated that PAβN and polyamino-isoprene derivatives permeabilized bacterial membranes and inhibited the efflux pump, thus augmenting the accumulation of antibiotics in pathogens [100,101]. Nevertheless, some EPIs enter the outer membrane by impairing rather than penetrating the outer membrane proteins, resulting in the over-augmented permeability of the membrane. The increased permeability of bacterial membrane can not only enable the increased influx of antibiotics but also be sufficient to induce bacterial lysis [99], indicating the potential off-target effects and cytotoxicity in clinical application. Therefore, the exploration of EPIs that can penetrate rather than impair the bacterial membrane may offer less cytotoxicity and have broad application prospects in controlling antibiotic resistance.

## 6. Conventional and Synthetic EPIs

It has been documented that conventional EPIs can impede the function of efflux pumps by various mechanisms and restore the efficacy of antimicrobial agents. Many synthetic EPIs have been identified and extensively investigated, such as PAβN, CCCP, 1-(1-Naphthylmethyl)-piperazine (NMP), and MBX2319 (Table 3). As a peptidomimetic compound, PAβN has been demonstrated to inhibit diverse efflux transporters and augment the efficacy of antibiotics such as macrolides, fluoroquinolones, and oxazolidinones [102]. Several mechanisms were involved in the inhibitory effects, including the competitive inhibition of antibiotics [103,104], the downregulation of efflux-related genes [105], and the adjustment of membrane permeability [101]. However, PAβN was associated with cytotoxicity towards mammalian cells, which limited its potential for clinical use. CCCP has been characterized as a broad-spectrum efflux inhibitor that suppresses the activity of most efflux pumps by interfering with ATP synthesis and electrochemical gradients based on PMF [106]. Previous studies reported that CCCP potentiated the antimicrobial activity of imipenem and cefepime against clinical strains of *A. baumannii* [107]. Additionally, CCCP can also induce metabolically inactive cells, leading to synergistic effects with antibiotics [108]. Notably, cellular toxicity was described in many studies, which limited the development for clinical application.

As one of the main aryl piperazine compounds, NMP can effectively reverse antibiotic resistance in *E. coli* and increase their susceptibility to fluoroquinolones [109]. Evidence showed that NMP was capable of restoring the substrate activity of RND transporters via interference with the functional assembly of efflux pumps [30,89]. Due to their similarity to serotonin agonists, arylpiperazine compounds may be harmful to mammalian cells. MBX2319 is a synthetic pyrazolopyridine that can augment the efficacy of various antibiotics such as ciprofloxacin, levofloxacin, benzoxicillin, and chloramphenicol against bacteria [136]. A study reported that MBX2319 decreased the MIC of levofloxacin by 4 times in *E. coli*, resulting from the competitive inhibition and blockage of access to the substrate binding sites [110]. In addition, MBX2319 did not exhibit bactericidal activity and only combat bacteria expressing efflux pumps. Nonetheless, the cytotoxicity to cells observed in research made it unsuitable for EPI in the clinic [136]. Currently, the extensive cytotoxicity of conventional EPIs impedes their clinical applications and makes them suited for research purposes in the laboratory only. Therefore, it is urgent to explore novel and safe EPIs from various sources in response to growing antibiotic resistance.

## 7. Discovery of Novel Natural EPIs

In general, most plants can generate certain beneficial molecules to protect themselves against invasive bacteria. These compounds mainly include flavonoids, essential oils, and alkaloids, which have been identified as potential EPIs (Table 3). Flavonoid compounds are derived from plant extracts and widely distributed throughout the leaves, flowers, roots, and fruits of various plant species. These compounds are renowned for their diverse biological properties, including anticancer, antioxidant, antimicrobial, antiallergic, and anti-inflammatory activities [137,138,139]. According to the relevant reports, certain flavonoid compounds have exhibited effectiveness in inhibiting efflux pump activity and enhancing the efficacy of antibiotics. As an illustration, the isoflavone biochanin A exhibited inhibitory activity towards the extrusion of EtBr facilitated by efflux pumps in *Mycobacterium smegmatis* [140]. Similarly, silybin suppressed the expression of NorA (36%) and qacA/B (49%) in methicillin-resistant *S. aureus* (MRSA), reinstating the susceptibility of MRSA to antibiotics [111]. In addition, boeravinone B has also been demonstrated to enhance the efficacy of ciprofloxacin on *S. aureus* and inhibit biofilm formation [112]. Previous studies have elucidated that related mechanisms of flavonoids are involved in the inhibition of efflux pumps. These mechanisms mainly include gene expression regulation, energy support impediment, and cell membrane damage induction. For instance, curcumin, derived from the rhizome of turmeric, can effectively reverse the TetK overexpression in *E. coli* and restore tetracycline activity [93]. Likewise, luteolin is capable of inhibiting MsrA efflux pumps by simultaneously decreasing *msrA* gene expression and blocking energy acquisition [54]. Baicalin derived from the roots of *Scutellaria baicalensis Georgi* can interfere with ATP synthesis involved in the MsrA function and regulate the biofilm formation and *agr* system [74]. In addition, owing to the lipophilic property of bacterial membranes, the lipophilic or hydrophobic flavonoid compounds can readily invade the bacterial membranes, leading to membrane damage, disruption of PMF, and the hampered activity of efflux pumps [141,142]. Thus, flavonoid compounds may hold promise as potential EPIs in combination with antibiotics for the treatment of bacterial infections. Additionally, it has also been observed that many flavonoid compounds exhibit low or no toxicity when compared to other existing EPIs [54,143].

As plant secondary metabolites, essential oils exhibit numerous pharmacological properties, including anti-inflammatory, anti-cancer, insecticidal, and antimicrobial effects [144,145]. Recent studies have demonstrated that essential oils can inhibit efflux pumps in MDR pathogens and potentiate antibiotic efficacy. For example, Cirino et al. [113] reported that *Origanum vulgare* L. essential oil effectively inhibits TetK efflux proteins and enhances the activity of tetracycline against *S. aureus* [113]. *Origanum Majorana* L. essential oil can effectively inhibit the activity of efflux pumps and biofilm formation in *S. aureus* and *E*. *coli* [119]. The essential oil derived from *Nigella sativa* can significantly reduce the MIC of antibiotics and inhibit the biofilm formation by *S. aureus* [120]. Similarly, *Cuminum cyminum* L. essential oil has been demonstrated to inhibit the NorA activity, QS system, and production of PIA involved in biofilm formation [121]. Furthermore, certain essential oils have been shown to possess direct antibacterial properties. A study evaluated 20 natural essential oils and documented their potent antibacterial activity against *Streptococcus mutans* [146]. Previous studies have documented that essential oils exhibit the capability to augment membrane permeability, disrupt cell membranes, and decrease ATP synthesis, thereby enabling the inhibition of efflux pump function and the accumulation of antibiotics [147,148]. Hydrophobic essential oils such as tea tree oil, thymol, and carvacrol can impair the integrity of the cell membrane, resulting in significant ion leakage and the suppression of efflux activity [115,116]. Mustard and oregano can disturb the equilibrium between extracellular and intracellular ATP by disintegrating the cell membrane, ultimately resulting in decreased ATP utilization and efflux activity [149,150]. Significantly, essential oils possess inhibitory effects against pathogens and are less prone to developing resistance due to their complex composition [151,152]. Nevertheless, the toxicity of essential oils is due to the presence of various constituents. Additionally, the stability of essential oils may be affected by environmental factors, leading to potential decomposition [153].

Alkaloids are regarded as crucial therapeutic agents for human health. They have been discovered in diverse natural sources and offer a wide range of pharmacological benefits, including the antioxidant properties of CZK and Berberine [154,155], the anticancer effects of meleagrin and oxaline [156], and the hypolipidemic effects of jatrorrhizine and palmatine [157,158]. Currently, several studies have explored the antimicrobial impact of alkaloids on bacteria. As an early natural EPI, reserpine was extracted from the roots of *Rauwolfia vomitoria* or *Rauvolfia serpentina*. Studies have shown that reserpine can target various efflux pumps, including BmrA, NorA, TetK, and PatA/B, as well as augment antibiotic activity [159]. Specifically, reserpine is capable of decreasing the efflux of tetracycline in *B. subtilis* through the interaction with Bmr transporters [123]. It can also interfere with the PMF and disrupt the NorA in *S. aureus*, resulting in diminished antibiotic resistance to norfloxacin [124]. Notably, the induction of neurotoxicity has been verified in reserpine, which may be more appropriate for inhibition research instead of clinical utilization. In addition, tomatidine has demonstrated anti-virulence properties in *S*. *aureus* and the capacity to enhance the activity of aminoglycoside antibiotics [160]. Capsaicin is capable of suppressing the activity of the NorA pump and the invasiveness of S. aureus [127]. Plant-derived alkaloids have been found to exert inhibitory effects on bacteria through a variety of direct and indirect mechanisms. These mechanisms include the induction of bacterial death by causing intracellular content leakage [161], targeting protein kinase enzymes [162], and inducing DNA damage [163]. Alkaloids have also been identified as EPIs that regulate gene expression and maintain antibiotic concentration. As an illustration, jatrorrhizine can effectively reduce the expression of NorA at the mRNA level and impede the antibiotic resistance of MRSA [126]. Moreover, columbamine, a protoberberine alkaloid, has demonstrated the capacity to interfere with ATP synthesis and impact the formation of proton electrochemical gradients [98]. Nevertheless, there is insufficient literature available currently on this topic, as many experiments solely describe the inhibition of efflux pumps by alkaloids without illuminating the precise mechanisms involved.

In addition to these EPIs derived from plants, recent years have witnessed the emergence of additional microbial-derived extracts that exhibit comparable inhibition of efflux systems. For instance, venturicidin A extracted from soil *Actinomycetes* has been found to impede ATP synthesis, disrupt proton gradients, and subsequently lead to the accumulation of antibiotics in MRSA, *P. aeruginosa*, and *Enterococcus* [130,131]. EA-371α and EA-371δ are the fermentation extracts generated by *Streptomyces* MF-EA-371-NS1. They possess the ability to downregulate the gene expression of MexAB-OprM of *P. aeruginosa* PAM1032 [132]. Similarly, microbe-derived 2-(2-Aminophenyl) indole (RP2) and ethyl 4-bromopyrrole-2-carboxylate (RP1) have been demonstrated to effectively interact with efflux transporters and reverse the bacterial resistance to multiple antibiotics [133,134]. These two compounds can also exhibit great post-antibiotic effects (PAEs) and minimal cytotoxicity and side effects, showing great promise as EPIs in application [133,134]. Hence, numerous additional origins of EPIs such as natural extracts warrant further investigation. Regarding these novel EPIs, many advantages have been identified in utilization such as lower cytotoxicity, enriched inhibition mechanisms, less development of EPI resistance, and distinct inhibitory efficacy.

**Figure 4:**
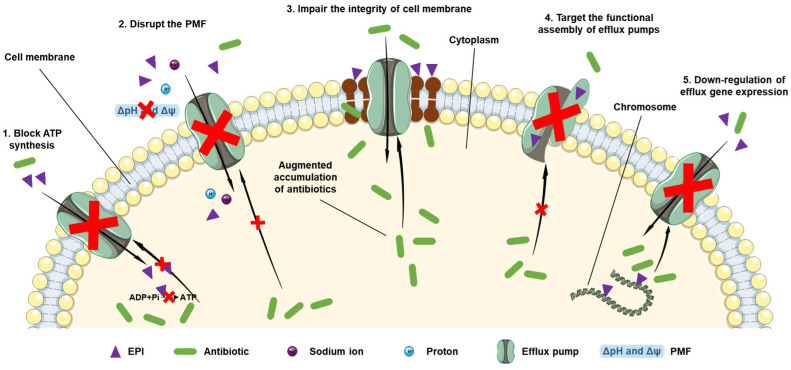
Inhibitory mechanisms of EPIs. 1: Blockage of adenosine triphosphate (ATP) synthesis to inhibit efflux. 2: Disruption of proton motive force (PMF) to suppress transport. 3: Increased concentration of intracellular antibiotics mediated by impairing the integrity of cell membrane. 4: Targeting the functional assembly of efflux pumps to suppress efflux. 5: Downregulating the expression of efflux genes to modulate functional efflux. ΔpH: transmembranepH gradient; Δψ: electrical membrane potential component.

In summary, the non-competitive inhibitory mechanisms of EPIs can be primarily divided into five different types: (i) blocking the synthesis and support of ATP; (ii) disrupting the ion gradients and PMF; (iii) impairing the membrane integrity; (iv) damaging the assembly of efflux pumps; (v) suppressing the expression of efflux genes (Figure 4). As previously mentioned, EPIs should refrain from becoming the substrates of efflux systems owing to the further evolution of drug resistance. Except for the competitive inhibition, the above five non-competitive inhibitory mechanisms exhibit great promise to develop into the excellent direction of EPIs. In addition, there are still several additional aspects meriting attention, including the appropriate pharmacokinetic profile, the lowest possible toxicity index, relative stability in utilization, and sufficient commercial value. Therefore, despite the achievement of massive progress in EPI research, the clinical translational research of EPIs still requires overcoming various challenges.

## 8. Concluding Remarks

In conclusion, the emergence of MDR pathogens has imposed mounting challenges on contemporary clinical environments. Antibiotic resistance mechanisms such as efflux pumps pose significant obstacles to developing novel antibiotics and their alternatives. Fortunately, the application of EPIs in combination with antibiotics has partially reduced the burden of antibiotic resistance. As a crucial source of bioactive molecules, natural plants have great promise for the discovery and development of a variety of effective EPIs. These EPIs have been demonstrated to exhibit multiple mechanisms, lower cytotoxicity, and less off-target effects in utilization. Many studies have been conducted to evaluate the efficacy of natural EPIs. However, the clinical results are still insufficient and require more verification. Notably, it seems to be a good alternative to develop natural EPIs instead of designing new antibiotics, which can be more economical and time-saving at a commercial level. However, it may also take a lot of sunk costs to find suitable EPIs for improving the clinical applicability of such EPIs. Additionally, the application of combination therapy has presented new challenges in this field. It is essential to manage and design appropriate treatment options to control antibiotic-resistant bacteria. Present studies have revealed that MDR pathogens can utilize compensatory mechanisms to counteract the inhibition of antibiotic potentiators. These mechanisms in question mainly manifest as the relatively stable antibiotic resistance reinforced by other known or unknown efflux pumps under combination treatments, resulting in incomplete inhibition and facilitating the emergence of multidrug resistance in bacteria. Consequently, it is crucial to undertake a comprehensive investigation of the structural and functional properties of EPIs for effectively controlling MDR pathogens. Moreover, a multitude of EPIs have demonstrated inhibitory efficacy towards antibiotic resistance of pathogens, although only one has received clinical approval. Hence, further comprehensive and in-depth research is necessary to facilitate their clinical transformation and utilization in the future in light of the intricate mechanisms, cytotoxicity, and pharmacokinetics of EPIs. The utilization of a combination therapy comprising antibacterial agents and EPIs has the potential to extend the lifespan of current agents and enhance their efficacy in addressing the challenges posed by the post-antibiotic era.

## Figures and Tables

**Table 1:** Major efflux pumps and corresponding regulators in bacteria.

Efflux Pump Family	Efflux Pump Regulator	Strain	Substrate	Reference
ABC (PATA/B)		*Streptococcus pneumoniae*	Ciprofloxacin, levofloxacin, and norfloxacin (hydrophilic fluoroquinolones)	[26]
ABC (MacAB-TolC)	BaeSR (−)	*Escherichia coli*	Lipopolysaccharides, polypeptide virulence factors, and macrolides	[27,28]
MFS (Tet38)	TetR21, MgrA (−)	*Staphylococcus aureus*	Glycerol-3-phosphate, fosfomycin, tetracycline, and certain unsaturated fatty acids	[29]
MFS (NorA)	NorR (+) MgrA (−)	*Staphylococcus aureus*	Fluoroquinolones, reserpine, dyes, pentamidine, phenothiazines, and omeprazole	[30]
MFS (QacA)	QacR (−)	*Staphylococcus aureus*	Bisbiguanides, quaternary ammonium compounds (QACs), diamides, and aromatic dyes	[31]
MFS (KpnGH)		*Klebsiella pneumoniae*	Detergents, cationic dyes, bile salts, and antiseptic chemicals	[32]
RND (AcrAB-TolC)	RamA, AcrR (+) MarR, SoxR (−)	*Escherichia coli*	Tetracycline, levofloxacin, chloramphenicol, norfloxacin, bile salts, organic solvents, fatty acids, and dyes	[33,34]
RND (MexAB-oprM)	BrlR, CpxR (+) mexR, nalD (−)	*Pseudomonas aeruginosa*	β-lactams, chloramphenicol, fluoroquinolones, macrolides, novobiocin, tetracycline, trimethoprim, detergents, organic solvents, and dyes	[35,36]
RND (FarE)	farR (−)	*Staphylococcus aureus*	Linoleic acid, fatty acid, and rhodomyrtone	[37]
RND (AdeABC)	AdeRS (−)	*Acinetobacter baumannii*	Aminoglycosides, β-lactams, chloramphenicol, erythromycin, tetracyclines, and EtBr	[38,39]
RND (AdeFGH)	AdeL (−)	*Acinetobacter baumannii*	Clindamycin, fluoroquinolones, and tigecycline	[40]
RND (AdeIJK)	AdeN (−)	*Acinetobacter baumannii*	β-lactam, fluoroquinolones, tetracyclines, tigecycline, lincosamides, rifampin, chloramphenicol, co-trimoxazole, novobiocin, and fusidic acid	[41]
MATE (PmpM)		*Pseudomonas aeruginosa*	Tetraphenylphosphonium chloride, acriflavine, EtBr, benzalkonium chloride, and fluoroquinolones	[42]
MATE (MepA)	mepR (−)	*Staphylococcus aureus*	Tigecycline, hydrophilic fluoroquinolones, dyes, and fungicides	[43]
SMR (EmrE)		*Escherichia coli*	Benzalkonium, EtBr, tetraphenylphosphonium, methyl viologen, betaine, and choline	[44]
SMR (KpnEF)	CpxR (+)	*Klebsiella pneumoniae*	Erythromycin, ceftriaxone, tetracycline, cefepime, rifampin, SDS, EtBr, chlorhexidine, benzalkonium chloride, triclosan, and acriflavine	[45]
SMR (QacC)		*Staphylococcus aureus*	Quaternary ammonium compound, chlorhexidine, and EtBr	[46,47]
SMR (EbrAB)		*Bacillus subtilis*	Cationic lipophilic dyes, including safranin O, pyronine Y, EtBr, and acriflavine	[48]
PACE (AceI)	AceR (+)	*Acinetobacter baumannii*	Proflavine, chlorhexidine, acriflavine, dequalinium, and benzalkonium	[49]

ABC: adenosine–triphosphate (ATP)-binding cassette superfamily; MFS: major facilitator superfamily; MATE: multidrug and toxic compound extrusion family; RND: resistance–nodulation–cell division superfamily; SMR: small multidrug resistance family; PACE: proteobacterial antimicrobial compound efflux family; EtBr: ethidium bromide.

**Table 2:** Potential efflux pumps involved in biofilm formation.

Biofilm Factor	Strain	Efflux Pump	Target Component	Function	Reference
EPS matrix	*Escherichia coli*	MFS (SetB)	Glucose	EPS matrix synthesis	[71]
	*E*. *coli*	ABC (YhdX)	L-amino acids	Biofilm stability	[72]
	*E*. *coli*	MFS (AraJ)	Arabinose	Bacterial aggregation	[65]
QS signals	*Pseudomonas aeruginosa*	RND (MexAB-OprM)	*N*-3-oxododecanoyl-l-homoserine lactone	Biofilm formation	[73]
	*Staphylococcus aureus*	ABC (MsrA)	*agrA* and *sarA*	Biofilm formation	[74]
	*P. aeruginosa*	RND (MexEF-OprN)	4-hydroxy-2-heptylquinoline (HHQ)	Quorum sensing quencher	[75]
Biofilm-associated genes	*Listeria monocytogenes*	ABC (Lm.G_1771)	SrtA, Dlt, and GntR	Biofilm-associated gene suppression	[76]
	*Enterobacteriaceae*	RND (AdeABC, AdeIJK)	*csuA/B*, *csuC*, and *fimA*	Adhesion and colonization interruption	[77]
	*Acinetobacter baumannii*	RND (AdeG)	*abaI*	AHL synthesis and transport	[78]
	*Salmonella typhimurium*	RND (AcrAB-TolC)	curli	Curli expression	[21]

EPS: extracellular polymeric substance; QS: quorum sensing; AHL: *N*-acyl homoserine lactones; ABC: adenosine–triphosphate (ATP)-binding cassette superfamily; MFS: major facilitator superfamily; MATE: multidrug and toxic compound extrusion family; RND: resistance–nodulation–cell division superfamily; SMR: small multidrug resistance family; PACE: proteobacterial antimicrobial compound efflux family.

**Table 3:** Synthetic and natural efflux pump inhibitors (EPIs).

Origin	Efflux Pump Inhibitor	Chemical Structure	Target Strain and Effective Substrate	Mechanism	Reference
Synthetic EPIs	PAβN	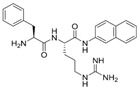	*P*. *aeruginosa* (MexAB-OprM transporters)—levofloxacin	Competitive inhibition, downregulation of efflux-related genes, and adjustment of membrane permeability	[17,99,101,105]
	CCCP	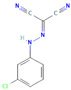	*A*. *baumannii*—imipenem and cefepime	Interference with ATP synthesis and electrochemical gradients	[106,108]
	NMP	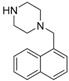	*Enterobacteriaceae*—oxacillin, linezolid, and rifampicin	Interruption of functional assembly of efflux pumps	[30,89,99,109]
	MBX2319	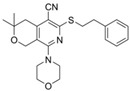	*E*. *coli*—levofloxacin	Competitive inhibition and blockage of access to the substrate-binding sites	[30,110]
Natural EPIs	Silybin	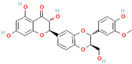	MRSA—ciprofloxacin and benzalkonium chloride	Downregulation of efflux-related genes	[111]
	Curcumin	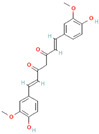	Clinical MRSA—ciprofloxacin	Downregulation of efflux-related genes	[93]
	Luteolin	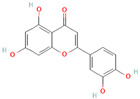	*T*. *pyogenes*—macrolides	Interference with ATP synthesis and downregulation of efflux-related genes	[54]
	Boeravinone B	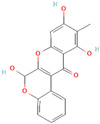	*S. aureus*—ciprofloxacin, EtBr	Interaction with the active sites of efflux pumps	[112]
	Baicalin	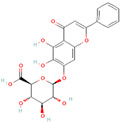	*S. saprophyticus*—EtBr	Interference with ATP synthesis	[74]
	*Origanum vulgare* L. EO		*S*. *aureus*—tetracycline	Downregulation of efflux-related genes	[113]
	*C. ambrosioides L.* EO		MRSA—tetracycline and ethidium bromide	Disruption of the proton transport and adjustment of membrane permeability	[114]
	Thymol and carvacrol		Gram-negative bacteria—tetracycline and benzalkonium chloride	Impairment of membrane integrity and induction of ion leakage	[115,116,117]
	*Salvia fruticosa* EO		*S. aureus*—tetracycline	Downregulation of efflux-related genes	[118]
	*Origanum Majorana* L. EO		*S. aureus* and *E*. *coli*—EtBr	Not mentioned	[119]
	*Nigella sativa* EO		MRSA—tetracycline, ciprofloxacin, and EtBr	Downregulation of efflux-related genes	[120]
	*Cuminum cyminum* L. EO		*S. aureus*—EtBr	Induction of conformational changes in efflux pump structures	[121]
	Reserpine	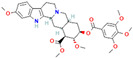	*B. subtilis*—tetracycline *S. aureus*—norfloxacin	Interference with proton gradients and interaction with efflux pump proteins	[122,123,124]
	Berberine	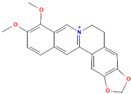	*P*. *aeruginosa*—imipenem	Downregulation of efflux-related genes	[125]
	Jatrorrhizine	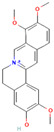	MRSA—norfloxacin	Downregulation of efflux-related genes	[126]
	Columbamine	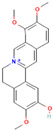	*E. coli*, *P. aeruginosa*, and *K. pneumoniae*—streptomycin, erythromycin, norfloxacin, ampicillin, ciprofloxacin, doxycycline, and chloramphenicol	Interference with ATP synthesis and generation of proton electrochemical gradients	[98]
	Capsaicin	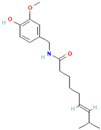	*S. aureus*—ciprofloxacin, EtBr	Docking to the active site	[127]
	Conessine	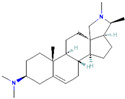	*P*. *aeruginosa*—cefotaxime, levofloxacin, tetracycline, erythromycin, novobiocin, and rifampicin	Competitive inhibition and/or blockage of access to the substrate-binding sites	[128]
	Catharanthine	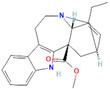	*P*. *aeruginosa*—tetracycline and streptomycin	Docking to the active site	[129]
	Venturicidin A	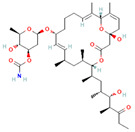	Aminoglycoside-resistant MRSA—gentamicin	Interference with ATP synthesis and proton gradients	[130,131]
	EA-371α EA-371δ	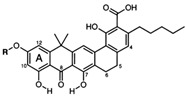 EA-371α 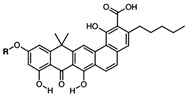 EA-371δ	*P*. *aeruginosa*—levofloxacin	Downregulation of efflux-related genes	[132]
	2-(2-Aminophenyl) indole (RP2)	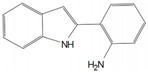	*S. aureus*—ciprofloxacin, tetracycline and erythromycin	Blockage of active site/channel	[133]
	Ethyl 4-bromopyrrole-2-carboxylate (RP1)	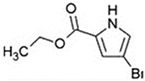	*E. coli* and *P. aeruginosa*—cloxacillin, ceftazidime, chloramphenicol, ciprofloxacin, erythromycin, levofloxacin, piperacillin, tetracycline, and tigecycline	Competitive inhibition	[134]
	3,4-dibromopyrrole-2,5-dione	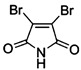	*E*. *coli*—ciprofloxacin, levofloxacin, kanamycin, erythromycin, piperacillin, tetracycline, and chloramphenicol	Not mentioned	[135]

PAβN: phenylalanyl arginyl β-naphthylamide; CCCP: carbonyl cyanide-m-chlorophenylhydrazone; NMP: 1-(1-Naphthylmethyl)-piperazine; MRSA: methicillin-resistant *Staphylococcus aureus*; EtBr: ethidium bromide; EO: essential oil; ATP: adenosine triphosphate.

## Data Availability

Not applicable.
